# Toughening of a Carbon-Fibre Composite Using Electrospun Poly(Hydroxyether of Bisphenol A) Nanofibrous Membranes Through Inverse Phase Separation and Inter-Domain Etherification

**DOI:** 10.3390/ma4111967

**Published:** 2011-11-02

**Authors:** Kevin Magniez, Thomas Chaffraix, Bronwyn Fox

**Affiliations:** 1 Institute of Technology Research and Innovation, Deakin University, Victoria, Waurn Ponds 3217, Australia; E-Mails: tchaffra@deakin.edu.au (T.C.); bronwyn.fox@deakin.edu.au (B.F.); 2 École Nationale Supérieure des Arts et Industries Textiles (ENSAIT), Roubaix 59056, France

**Keywords:** poly(hydroxyether of bisphenol A), nanofibres, fracture toughness, delamination, electro-spinning

## Abstract

The interlaminar toughening of a carbon fibre reinforced composite by interleaving a thin layer (~20 microns) of poly(hydroxyether of bisphenol A) (phenoxy) nanofibres was explored in this work. Nanofibres, free of defect and averaging several hundred nanometres, were produced by electrospinning directly onto a pre-impregnated carbon fibre material (Toray G83C) at various concentrations between 0.5 wt % and 2 wt %. During curing at 150 °C, phenoxy diffuses through the epoxy resin to form a semi interpenetrating network with an inverse phase type of morphology where the epoxy became the co-continuous phase with a nodular morphology. This type of morphology improved the fracture toughness in mode I (opening failure) and mode II (in-plane shear failure) by up to 150% and 30%, respectively. Interlaminar shear stress test results showed that the interleaving did not negatively affect the effective in-plane strength of the composites. Furthermore, there was some evidence from DMTA and FT-IR analysis to suggest that inter-domain etherification between the residual epoxide groups with the pendant hydroxyl groups of the phenoxy occurred, also leading to an increase in glass transition temperature (~7.5 °C).

## 1. Introduction

Owing to their exceptional engineering properties, carbon fibre reinforced composites (CFRC) belong to a class of advanced materials which have witnessed significant progress in recent years. More recently, CFRC have rapidly become an alternative replacement to other traditional materials and have found applications in the automotive and aerospace sectors. The new Boeing 787 is a modern example of technological achievement that utilises up to 50 weight percent of lightweight composite materials [1]. However, when exposed to cyclic mechanical and thermal loading, CFRC are prone to a wide variety of damage modes such as fibre breakage, debonding, transverse-ply cracking and delamination; the latter being the most prominent. 

The lack of toughness at the fibre/matrix interface, stemming from the inherent brittleness of thermosetting resins, has been associated with the issue of delamination in composites. As a result, a substantial amount of research has been dedicated over the past two decades or so to improving the toughness of thermosetting resins. This has been realised through the development of novel resin systems involving the use of elastomers and thermoplastics as toughening agents [2,3]. Interlayer toughening described as the inclusion of discrete layers of a secondary material, in film, fibrous or particulate form, in the inter-plies region has also been extensively explored in the 80’s [4,5,6,7,8,9,10]. The concept of interleaved composites was introduced by Cytec (Formerly American Cyanamid) with the CYCOM HST-7 product, achieving improved toughness through the introduction of a discrete high toughness and high strain epoxy layer on one side of the pre-impregnated material. Other commercial examples of commercial toughened pre-preg systems are from Cytec are CYCOM 985 and CYCOM 1808 [11,12] and Torayca T800H/3900-2 which was qualified as the first composite materials for primary structures of Boeing Material Specification (BMS 8-276), later on utilised in primary structures of the Boeing 777. T800H/3900-2 contains a heterogeneous interlayer of fine thermoplastic particulates of amorphous polyamide. 

Despite the comprehensive amount of research available on the electrospinning of thermoplastic polymers into nanofibres, there has been comparatively little research carried out (at least in the open literature) on the interlayer toughening of composites using electrospun nanofibrers. This innovative approach could potentially become of importance since the design of low to medium volume electrospinning equipment is now commercially available (see for instance Elmarco's Nanospider). The interlayer toughening of composites using electrospun nanofibrers was patented by Dzenis [13] in 2001. It is only very recently that this approach has been reported in the scientific literature. Li *et al*. showed an enhancement of the mode I resistance to delamination of a CFRC using polysulfone (PSF) nanofibres [14,15]. Jin *et al*. also published some increases by controlling the diameter and the interlayer thickness of electrospun Polyetherketone Cardo (PEK-C) nanofibres [16] while Liu found no variation using epoxy [17] nanofibres. Sihn investigated the use of electrospun polycarbonate, poly(phenylene oxide) and polystyrene nanocomposites based on carbon nanotubes [18]. The introduction of the nano-interlayers into Cycom AS4/977 was found to suppress the microcracking and delamination damage.

**Figure 1:**
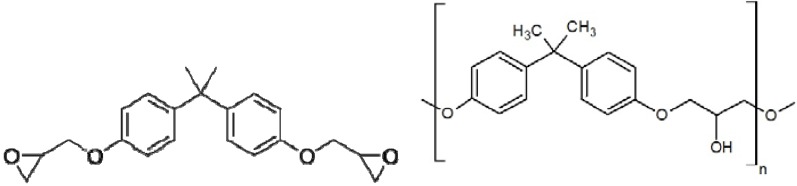
Chemical structure of diglycidyl ether of bisphenol-A (DGEBA, **left**) and poly(hydroxyether of bisphenol A) (phenoxy, **right**).

In a previous paper [19] we explored the interlaminar toughening of an aerospace grade CFRC through the incorporation of electrospun polyvinylidene fluoride (PVDF) nanofibrous in a simple one step process. The improved plastic deformation at the crack tip after inclusion of the nanofibres was directly translated to a 57% increase in the mode II interlaminar fracture toughness (in-plane shear failure) of the resulting composites. The mode I (opening failure) results however were found to be slightly lower as a result of complex micromechanisms of failure and polymorphic behaviour of the PVDF. This work has extended on this concept and looked at phenoxy, a well-known thermoplastic of chemical structure similar to that of the typical diglycidyl ether of bisphenol-A (DGEBA) epoxy resin (Figure 1), as a potential interlaminar toughening agent.

Increases in K_IC_ and weight impact fracture toughness of the epoxy after blending with phenoxy [20,21] has been previously reported and these improvements have been shown to arise from the phase separation mechanisms during curing. Phenoxy in the form of continuous multi-filament fibre was commercialised by EMS-Griltech as a dissolvable stitching yarn for non-crimp fabrics (NCF) under the trademark MS Grilon. More recently phenoxy fibres have appeared in the form of non-woven interleaf binding fibrous mats (approximately 50 microns fibre diameter, 20 g/m^2^). Beier *et al*. showed that the mechanical performances (in terms of mode 1 and mode 2 interlaminar energy release rates and compression strength) of an infused non-crimp carbon fibre-reinforced composite were found to be marginally improved using either a stitched phenoxy yarn (150 dtex) or a non-woven phenoxy mat [22]. After introduction of 10 wt % phenoxy into the resin system (Hexflow RTM6) however a 7 °C decrease in Tg was observed as the density of the resin network became less dense. The phase separation mechanisms and interfacial interactions between the epoxy and the phenoxy were not discussed, and nor was their effect on the mechanism of failure during the various modes of loading. 

This paper aims to cover the gaps found in the literature and the interfacial interactions between the phenoxy and the epoxy matrix will be discussed. In addition, in order to enhance the epoxy/phenoxy interaction, the interlaminar toughening was explored through electro-spinning of phenoxy nanofibres. The fracture toughness results were correlated to both the micromechanisms of failure and micro-domain morphology of the epoxy/phenoxy interface. Finally, this article will discuss the effect of interlayer modification on the thermo-mechanical performance of the resulting composites and the changes in both properties and glass transitions will be interpreted.

## 2. Results and Discussion 

### 2.1. Morphology of the Phenoxy Nanofibrous Membranes

**Figure 2:**
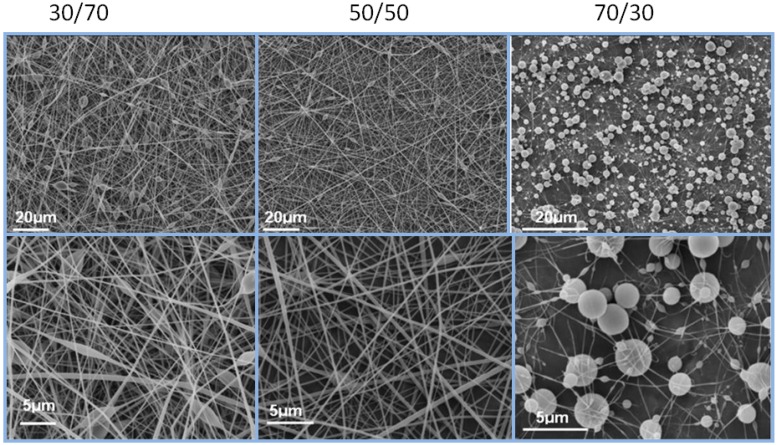
Effect of dimethylformamide (DMF)/tetrahydrofuran (THF) solvent volume ratios on the morphology of the electrospun phenoxy at a polymer concentration of 15% (increasing magnification from top to bottom).

To date, there is no available literature on the electrospinning of phenoxy. The selection of a suitable solvent was an important first step in order to successfully electrospin phenoxy into nanofibres. Phenoxy is miscible with THF, which is commonly used in electrospinning. However, it has been often found in the literature that THF is too volatile to electrospin nanofibers free of defects and as a result mixture of solvents have been utilised in order to decrease the volatility. In this work, we used various mixtures of THF and DMF at 30/70, 50/50 and 70/30 volume ratios. The SEM images displayed in Figure 2 show the effect of solvent volume ratios (DMF/THF) on the morphology of the electrospun phenoxy and one can noticed the differences in morphologies between the samples. The amount of beads seemed increase as the ratio of DMF also increased. At 70/30 (vol.%) only micron-size beads were present in the sample, with barely any visible nanofibres. At both 30/70 and 50/50 (vol.%), nanofibres were formed but the quality (in terms of both homogeneity and statistical distribution) of the nanofibres was found to be better at 30/70 (vol.%), and thus this ratio was selected for the rest of the study. 

**Figure 3:**
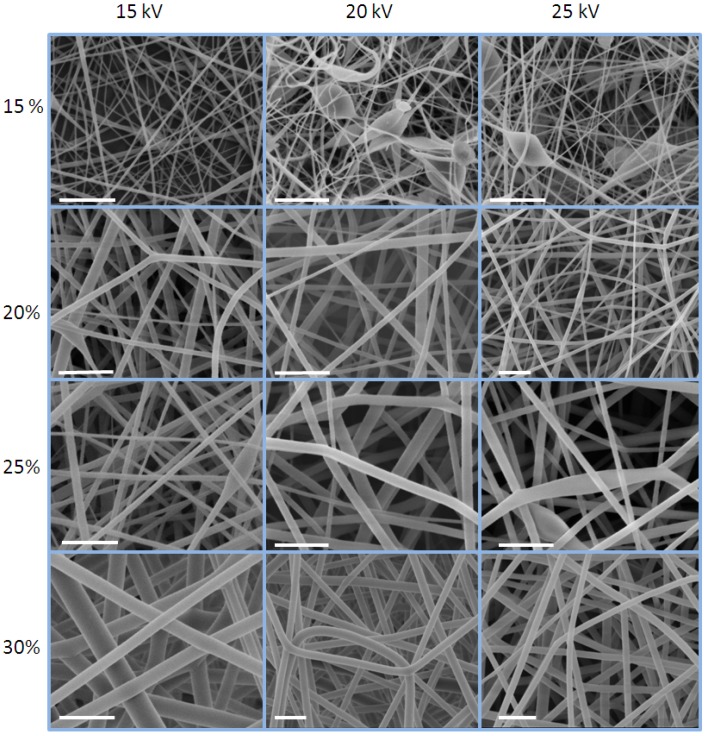
Effects of the phenoxy concentration (at a fixed DMF/THF volume ratio of 30/70) and voltages on the morphology of the electrospun nanofibres (scale bars are 5 microns).

The physical properties of the electrospun nanofibres such as fibre shape, diameter, surface morphology, or porosity are often strongly influenced by the electrospinning parameters [23]. This is well illustrated from the SEM images of the phenoxy nanofibres taken at various solution concentration (15–30 wt %) and various voltages (15–25 kV) (Figure 3). The presence of beads and inhomogeneities is noticeable at a phenoxy concentration 15 wt %. At higher concentrations, the nanofibres displayed minimum defects and random orientation. The homogeneity and the diameter of nanofibers produced seemed however to vary between samples. Increases in the concentration of the phenoxy induced an increase in diameter and conversely, increases in the voltages induced a decrease in diameter. Under optimum conditions of 25 kV and 30 wt %, the nanofibres had an average diameter of 909 ± 126 nm and were found to be very homogeneous. The phenoxy was electrospun directly onto the pre-impregnated woven material under these conditions. 

### 2.2. Double Cantilever Beam (DCB) Fracture Toughness (Mode I)

**Figure 4:**
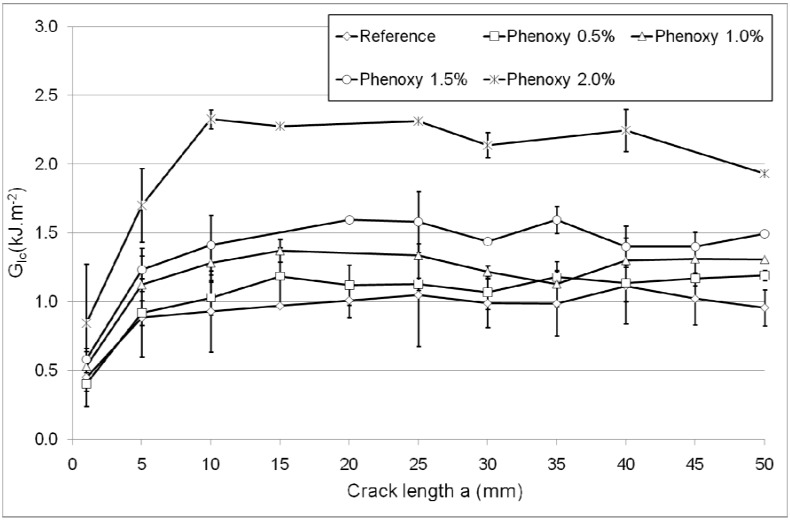
Fracture toughness (G_IC_) as a function of the crack length for the reference composite and composite modified with various weight percent of phenoxy nanofibres.

Evaluation of the fracture toughness in composite laminates is very important since the debonding between plies (referred to as interlaminar delamination) can significantly compromise the integrity of the structure. Interlaminar delamination often results from the formation and propagation of microcracks which are caused by thermal and mechanical stresses. The study of delamination in composites is often carried out by testing their fracture toughness in various modes of failure (namely opening, in-plane shear and out-of plane shear) [6]. The double cantilever beam (DCB) fracture toughness (mode I) represents the resistance of a material to delaminate in an opening mode of failure upon crack propagation (Figure 4). The mode I fracture toughness (referred to as G_IC_) results are summarised in Table 1. 

G_IC_ (initiation) is defined as the slope of the curves when the fracture toughness is growing monotonously and stands for the energy necessary to initiate the crack in the specimen. The G_IC_ (initiation) trends summarised in Table 1 indicate the amount of energy required to initiate the crack is increasing with increasing phenoxy content, and at 2 wt % of phenoxy, the G_IC_ (initiation) value has doubled. 

**Table 1:** Fracture toughness (mode I) results.

	G_IC_ (initiation)^1^ (kJ/m²)	G_IC_ (propagation)^2^ (kJ/m²)	G_IC (Max)_( kJ/m²)
Reference	0.1085	1.00 ± 0.05	1.12 ± 0.27
Phenoxy 0.5%	0.1285	1.13 ± 0.06	1.19 ± 0.04
Phenoxy 1.0%	0.1485	1.26 ± 0.09	1.34 ± 0.05
Phenoxy 1.5%	0.1629	1.46 ± 0.12	1.6 ± 0.15
Phenoxy 2.0%	0.2143	2.18 ± 0.13	2.31 ± 0.15

**Figure 5:**
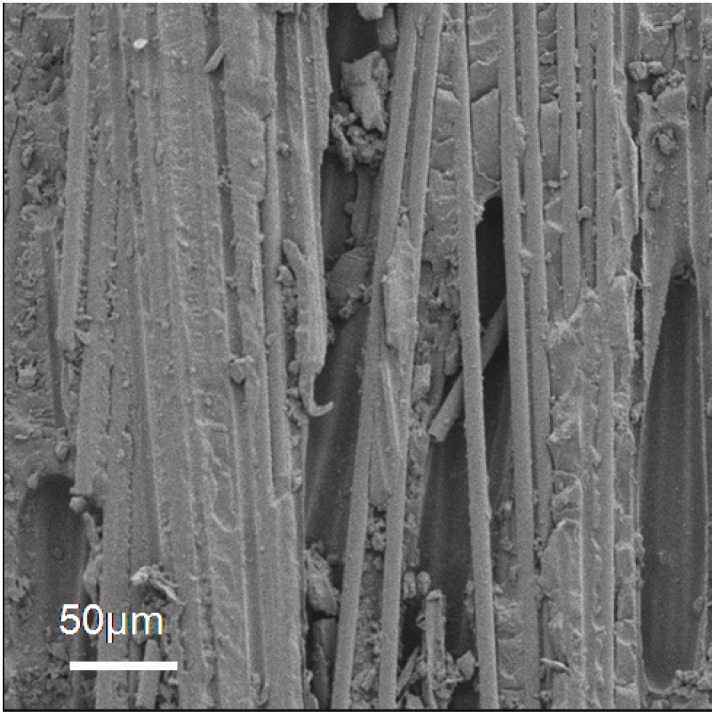
Scanning electron microscopy (SEM) fractography images of the reference sample after double cantilever beam (DCB) test showing a typical brittle interlaminar surface.

During propagation, the fracture toughness becomes more stable. The average propagation and maximum values of G_IC_ for each specimen are summarised in Table 1. It can be noted that the reference sample under-performed in comparison to the other samples and displayed a resistance to crack propagation with an average value G_IC_ of 1 kJ/m². Surface fractography analysis revealed a typical brittle interlaminar fracture surface (Figure 5) where interfacial debonding between the matrix and the carbon fibres appears to be the dominant failure mode. 

**Figure 6:**
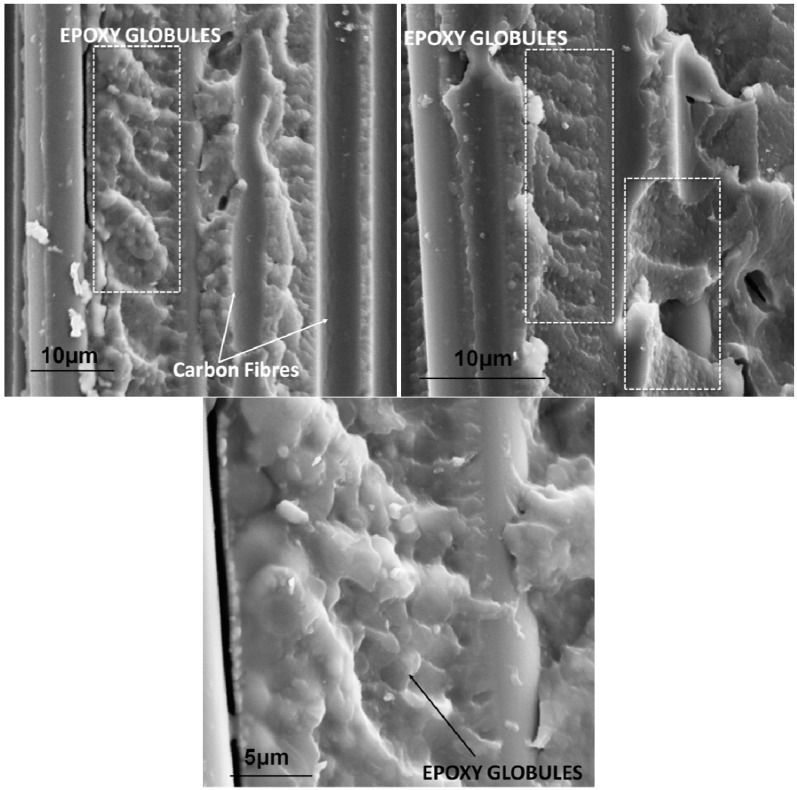
Fractography images of the sample reinforced with 2 wt % phenoxy after DCB test. The images show a reverse morphology at the fibre/matrix interface (note that the phenoxy phase was chemically etched with DMF).

The inclusion of phenoxy nanofibres in the interlayer region increased the average resistance to crack propagation G_IC_ by up to 118%. This trend is consistent with the recently reported literature showing various levels of improvement (15 to 280%) in carbon fibre epoxy composite using polybenzimidazole (PBI) and polysulfone electrospun nanofibres [13,14,15]. The toughening of epoxy by polysulfone [14,15] and phenoxy [20,21] has been shown to arise from the reaction induced phase separation mechanisms. In this work, it was found that the epoxy phase was homogeneously ‘dispersed’ in the continuum of the minor phase phenoxy. Evidence for inversion of phase continuity between curing are well supported by the SEM surface fractography after DCB testing of the sample containing 2 wt % nanofibres (after removal of the phenoxy phase with DMF). The images show show the local formation of an inverse phase domain morphology characterised by epoxy globules (~2–5 μm) within the phenoxy-rich continuous phase (Figure 6). The mechanism for phase inversion that occurs during curing of phenoxy/epoxy blends are not well known but it is suggested that during curing the phenoxy rich nanofibrous interface melted and diffused through the epoxy phase to become the continuous phase. The location of these domains was however relatively sparse within the sample and the concentration of these domains was generally found to decrease with decreasing phenoxy wt % content (not shown here for brevity). It is suggested that the toughening effect has most probably been induced by cavitation or voiding micromechanisms of failure at the epoxy/phenoxy interphase.

### 2.3. Mode 2 Fracture Toughness and Interlaminar Shear Stress (ILSS)

**Figure 7:**
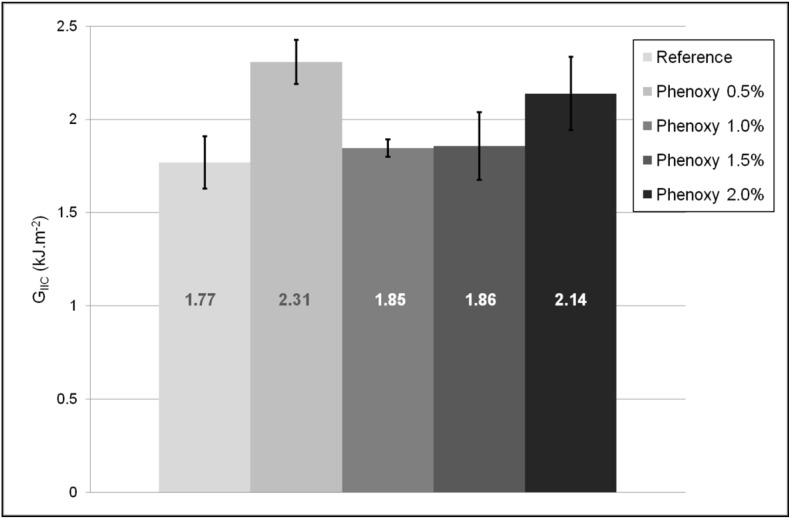
End notch fracture toughness (G_IIc_) results for the reference composite and a sample reinforced with 2 wt % phenoxy nanofibres in each interlayer region of the composite.

During the fracture toughness testing of composites using an end-notched flexure test (ENF), the micromechanisms of fracture are controlled by the in-plane shear plastic deformation of the matrix [6,24]. The mode II fracture toughness (referred to as G_IIC_) results are displayed in Figure 7. Effective interlayer toughening of composites has previously been reported using thermoplastic and thermosets films [6,10,25,26,27,28]. In this work, the introduction of electrospun phenoxy membranes improved the G_IIC_ values by approximately 30% but the results were skewed and not consistent across the various percent studied. For instance, the fracture toughness (G_IIC_) results for the composite modified with 1 and 1.5% phenoxy nanofibres remained very similar to the reference composite. This possibly indicates that the crack path transited from the mid-section of the nanofibrous interlayer region to the interfacial interlayer/resin region, but no evidence could be found in the cross section analysis to support this hypothesis. This type of behaviour has previously been described in interlayer toughened systems and it was attributed to the changes in micromechanisms of failure during loading [4,25]. 

**Figure 8:**
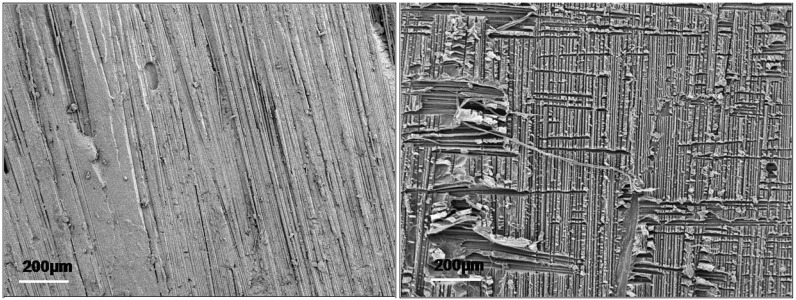
Fractography images of the reference sample (**left**) and sample reinforced with 2 wt % phenoxy (**right**) after end-notched flexure (ENF) test. (Note that only the 2% sample is shown for brevity).

The surface morphology of the reference sample (Figure 8) was found to be typically brittle and the shear plastic deformation of the matrix was noticeable from the presence of creases. 

It is well documented that improvements of the in-plane shear fracture toughness (mode II) are directly correlated to the ability of the matrix to undergo plastic deformation. In this work, it is proposed that a localised shear yielding micro-mechanism around the epoxy globules around the rich phenoxy continuous phase (which requires greater energy dissipation) at the crack tip may occur during loading and might have been contributing to some of the observed improvements as previously reported [21] (Figure 8). 

**Figure 9:**
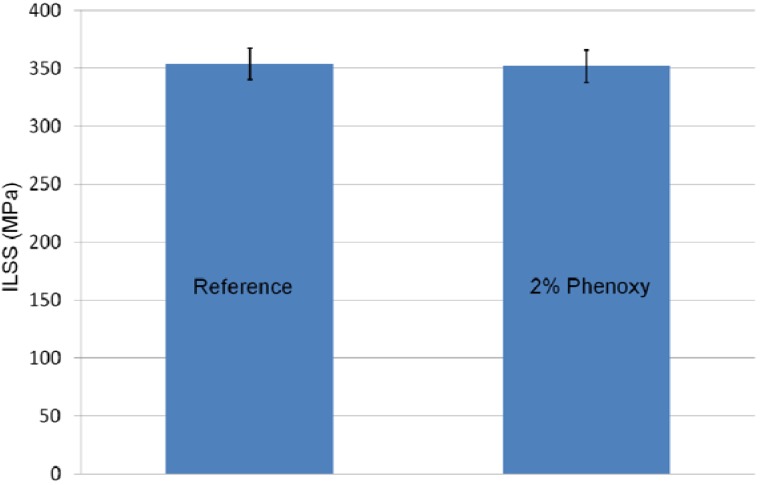
Interlaminar shear test (ILSS) results for the reference composite and composite modified with 2 wt % phenoxy nanofibres in each interlayer region of the composite.

The interlaminar shear stress (ILSS) results (Figure 9) of a sample containing 2% phenoxy nanofibrers between each layers (see experimental section for details) was similar to the reference sample. In other words, the interleaving method used in the work did not cause any decreases in effective in-plane stiffness as previously mentioned elsewhere [29]. 

### 2.4. Dynamic Mechanical Thermal Analysis

**Figure 10:**
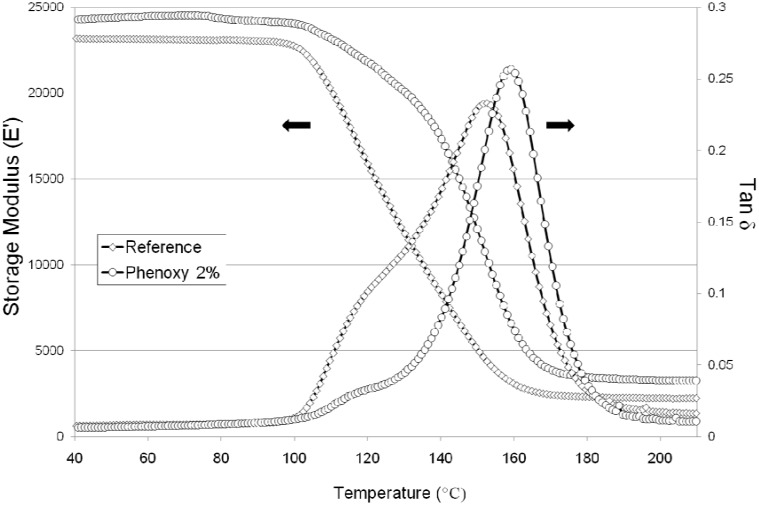
Storage modulus (E’) and loss tangent (tan δ) of the reference sample and a sample reinforced with 2 wt % phenoxy nanofibres in each interlayer region of the composite.

Figure 10 shows the storage modulus (E’) and loss tangent (tan δ) of the reference and a sample containing 2% phenoxy nanofibrers between each layer of the sample. This particular sample was produced in light of the mode I results in order to determine whether or not the presence of phenoxy would negatively affect the thermo-mechanical properties of the composite sample if present between each inter-ply region. It is important to point out at this stage that the increase thickness with and without the phenoxy was only approximately 70 microns (based on an average of 5 spots measurement per sample). 

Interestingly the thermo-mechanical properties (storage modulus and glass transition temperature Tg) of the composite sample reinforced with 2% phenoxy were found to be improved over the range of tested temperatures (30–250 °C). The measured increase in thickness of the laminate after introduction of the nanofibres was minimal (70 microns, reference being ~3.5 mm) hence the observed improvements could not possibly be associated with that. Looking into details at the tan δ curves, the visible broad shoulder in the reference sample at 120–130 °C is typical of residual epoxides which are often in excess in commercial formulations. The tan δ curve of the sample reinforced with 2 wt % phenoxy nanofibres was found to be much narrower. In addition, a small hump at approximately 110–120 °C could be observed, indicative of the presence of a phase separated phenoxy. The increase in T_g_ of approximately 6.6 °C supports evidence of further cross-linking reaction occurring in the later stage of cure. This is in line with the literature reporting inter-domain etherification between the residual epoxide groups and the pendant hydroxyl groups of the phenoxy in phase-separated systems (TGDDM/DDS/Phenoxy) [30]. 

**Figure 11:**
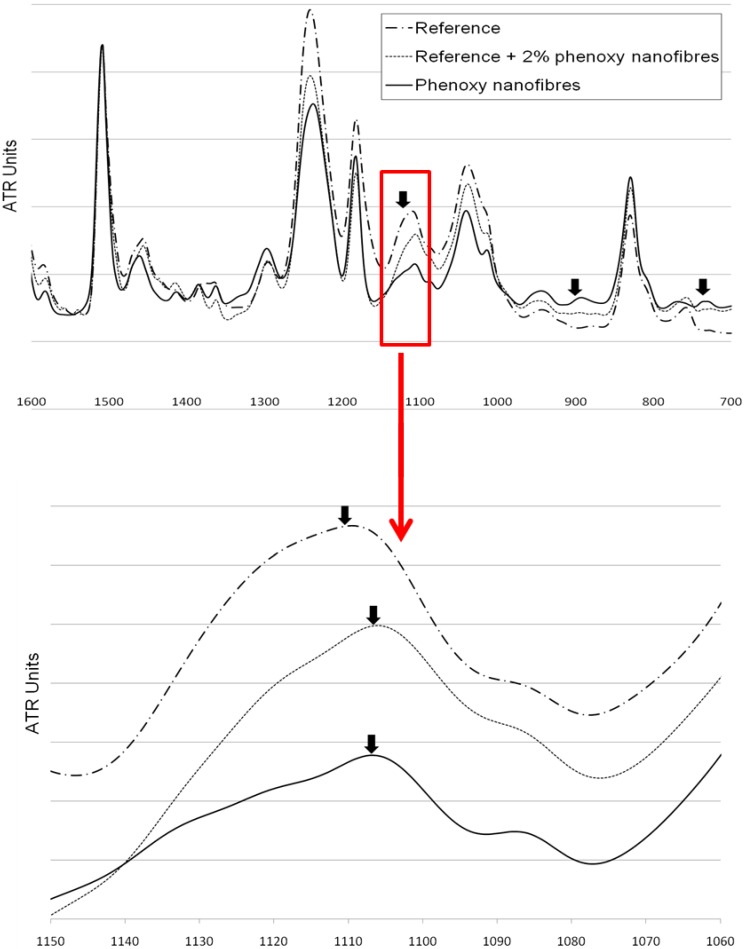
FTIR-ATR spectra of a reference sample and a sample cured with a phenoxy nanofibrous membrane on its top surface (accounting for a total of 2 wt %) between 700 cm^−1^ and 1600 cm^−1^.

We conducted FT-IR analysis to further elucidate some of the findings. A single ply of pre-preg material (10 mm × 10 mm) containing 2 wt % of phenoxy on the top surface was cured for 10 minutes at 150 °C. The remaining phenoxy was etched with DMF/THF (30/70 volume ratios) for 2 hours then ultra-sonicated. FT-IR spectra in ATR mode was analysed and compared with a reference sample cured under the same conditions without phenoxy (Figure 11). It can be noticed that the absorption bands of all spectra are very similar; this is not surprising as phenoxy is synthesised by reacting epichlorohydrin with bisphenol A and is chemically similar to Diglycidyl ether of bisphenol-A (DGEBA) epoxy resin, although it does not possess epoxide groups (Figure 10). After etching of the composite containing the phenoxy, some of the characteristic absorption bands at 736 cm^−1^ and 890 cm^−1^ suggested that the phenoxy has possibly reacted at the interface and some of the polymer chains were still present and attached on the top surface of the material. Moreover, the reference sample displayed characteristic bands of the asymmetrical stretching of C-O-C groups at 1,110 cm^−1^ [31]. However, a small shift of 7 cm^−1^ in the asymmetrical stretching vibration of C-O-C was observed (which corresponds to the stretching vibration of C-O-C in pure phenoxy), further emphasising the likeliness of interfacial reactions between phenoxy and epoxide.

## 3. Experimental Section

### 3.1. Materials

Carbon fibre reinforced composites were manufactured using Toray G83C (12K 2 × 2 twill 380 gsm) supplied by Toray Composites Tacoma, Washington USA. Poly (hydroxyether of bisphenol A) (referred to as phenoxy), with an average Mw = 40,000, was obtained from Aldrich Chemical Company, Inc.

### 3.2. Preparation of Phenoxy Nanofibrous Membranes by Electrospinning

Solutions containing 15 wt %, 20 wt %, 25 wt % and 30 wt % of phenoxy were prepared by dissolving pellets in 30/70 volume ratios dimethylformamide (DMF)/ tetrahydrofuran (THF) solvent with stirring for 3 hours at room temperature. The solutions were placed in a 5 mL medical syringe (0.8 mm needle diameter) connected with laboratory single jet system (KDS Syringe Pump) placed on a moving platform (velocity of 3 mm/s). The flow rate and the needle tip-to-collector distance were set to 1.5 mL/h and 15 cm, respectively. 

In the first stages of the work, we identified the optimum conditions for which phenoxy nanofibres could be formed with minimum defects and uniform diameters. The so-called optimisation process was achieved by electrospinning phenoxy directly onto a collector placed at a distance of 15 cm using voltages of 15 kV, 20 kV and 25 kV. The morphology of the nanofibres produced under these conditions was analysed under the SEM.

The phenoxy nanofibres used to reinforced the composites were produced by electrospinning directly onto a ply of Toray G83C ply that was stuck using conductive carbon tape onto a cylinder rotating (120 rpm, 26 m/min), using voltage of 25 kV and solution concentration of 30 wt % (*i.e.*, optimised conditions, see discussion section). The amount of phenoxy nanofibres electrospun onto the pre-preg material was controlled to 0.5 wt %, 1 wt %, 1.5 wt % and 2 wt % of the laminate mid-plane.

### 3.3. Composite Manufacture 

Five types of composites were produced for this work: a reference composite and four composites reinforced with approximately 0.5 wt %, 1 wt %, 1.5 wt % and 2 wt % in the laminates’ mid-plane of phenoxy nanofibrous membranes. A symmetric lay-up sequence [0/90] was achieved by stacking 8 plies of pre-impregnated G83C woven fabric on top of each other, which was then cured on an aluminum plate coated with a release agent. Since the fracture toughness was to be evaluated by allowing the crack to propagate only between the fourth and the fifth plies, the nanofibrous membrane were placed between those respective plies. A release film was inserted in one end of the laminates between the fourth and fifth plies as a pre-crack for the DCB and ENF tests. The thickness of all produced laminates was 3.5 ± 0.2 mm. 

De-bulking was performed every second ply for 10 min at room temperature to remove any entrapped air between the layers during lay-up. In addition, the plies containing the nanofibrous membranes were degassed for two hours in a vacuum oven to remove any excess solvent from the electrospinning. The laminates were vacuum bagged at 90 kPa overnight prior to and during cure. G83C is a fast curing epoxy-based resin system designed to cure in 10 minutes at 150 °C. The curing cycle employed in this work was a ramp from room temperature to 150 °C at approximately 2 °C/min, followed by a dwell for 10 minutes before cooling. 

Note that a small composite laminate (150 mm × 150 mm) reinforced with approximately 2 wt % phenoxy between each inter-ply region was also produced. The specimens cut from this laminate were used to evaluate the effect of the phenoxy on the thermo-mechanical properties (storage modulus and glass transition) and interlaminar shear stress (ILLS) properties.

### 3.4. Testing 

#### 3.4.1. Double Cantilever Beam (DCB) Fracture Toughness (Mode I)

**Figure 12:**
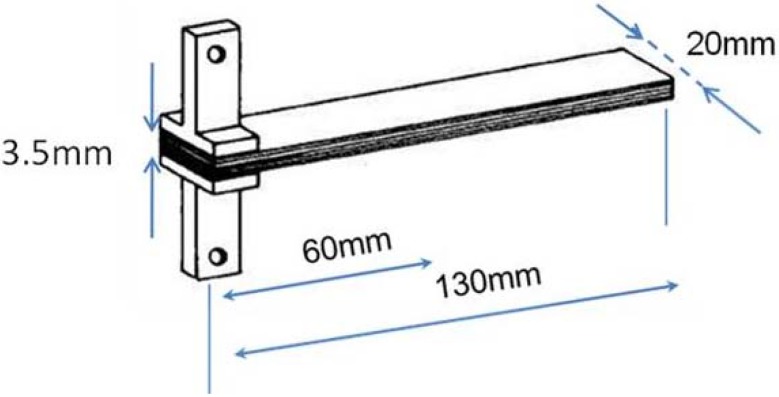
Sample geometry for double cantilever beam (DCB) fracture toughness test.

DCB testing was performed in the perpendicular direction to the length under a constant speed of 1 mm/min using 1kN load using a Lloyd tensile testing machine following the protocol for interlaminar fracture testing (European Structural Integrity Society Standard) [32]. The dimensions of the DCB test samples were approximately 130 mm × 20 mm × 3.5 mm with a pre-crack length of 60 mm. Five DCB specimens were cut from the laminates using a diamond saw. Aluminum blocks were bonded onto the two sides of the specimen end having the pre-crack (Figure 12). One side edge of the specimen was marked with white paint every 1 mm for the first 5 mm, and then, every 5 mm in order to facilitate the measurement of crack length. All samples were dried the day prior to testing (24 h, 70 °C). The width and the thickness of each specimen to the nearest 0.05 mm, at the mid-point and at 25 mm from both ends were measured, and their average values were calculated. The corrected beam theory described in the standard [32] was used to calculate G_IC_ values using the following Equation:

$$G_{IC}=\frac{3P\delta }{2B(a+|\Delta |)}$$

Where P is applied load, δ is displacement, B is specimen width, a is crack length, and |Δ| is the crack length correction factor, which is found experimentally by plotting the cube root of compliance, C^1/3^ as a function of a.

#### 3.4.2. End Notch Flexure (ENF) Fracture Toughness (Mode II)

**Figure 13:**
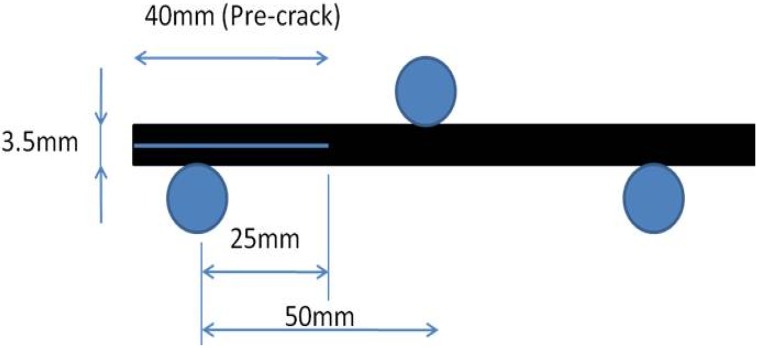
Sample geometry for end notch flexure double (ENF) fracture toughness test.

ENF testing was performed in a three-point bending mode using an Instron tensile testing machine with a crosshead speed of 1 mm/min following the protocol for interlaminar fracture testing (European Structural Integrity Society Standard) [33]. The dimensions of the ENF test samples were approximately 130 mm × 20 mm × 3.5 mm with a pre-crack length of 40 mm (Figure 13). The width and thickness of each specimen was measured to the nearest 0.025 mm at the midpoint and at 10 mm from each end. Five ENF specimens cut from the laminates using a diamond saw were used for this test. The direct beam theory described in the standard was used to calculate G_IIc_ using the following Expression [33]:

$$G_{IIc}=\frac{9a^{2}\text{P}\delta }{2B(2L^{3}+3a^{3})}$$

where a is the crack length, P is the load, δ is the displacement, B is the specimen width and L is the half span.

#### 3.4.3. Interlaminar Shear Test (ILSS)

The interlaminar shear test (ILSS) test was conducted in accordance with ASTM D2344 at a crosshead speed of 1 mm/min using a span to thickness ratio of 4 and length to thickness ratio of 6. All specimens were simply supported in a fixture and loaded at mid span. The ILSS was calculated as follows: ILSS = 0.75 /b*d, where represents the breaking load, b the width of specimen and d the thickness of specimen. Five specimens cut from the laminates using an Accutom fine cutting machine at a speed of 1 mm/s.

#### 3.4.4. Dynamic and Mechanical Analysis (DMTA)

Samples cut from the laminate of approximate dimensions of 60 mm (length) × 15 mm (width) × 3.5 mm (thickness) were used. Analysis was performed in a bending deformation mode using dual cantilever geometry at a frequency of 1 Hz. The experiments were carried out between 25 °C and 250 °C at a heating rate of 2 °C/min and a strain of 0.05%. The glass transition of the polymer blend was determined at maximum of loss tangent tan (δ) curve.

#### 3.4.5. Fourier Transform Infrared Spectroscopy (FTIR)

For this study, two small sections of a single ply of pre-preg material (10 mm × 10 mm), one for reference and one containing 2 wt % of phenoxy on its top surface, were cured for 10 minutes at 150 °C. In order to completely dissolve the remaining phenoxy, the materials were etched with DMF/THF (30/70 volume ratios) for 2 hours then ultra-sonicated for 10 minutes. 

Infrared data were collected on a Bruker Vertex 70 FTIR in attenuated total reflectance mode using 128 scans at 6 cm^−1^ resolution and between 600 cm^−1^ and 1600 cm^−1^. The spectra were collected on the top surface which contained the phenoxy nanofibres for each sample. The spectra were normalised to the band at 1515 cm^−1^ corresponding to the absorption of the aromatic ring vibration.

#### 3.4.6. Scanning Electron Microscopy (SEM)

The morphologies of nanofibrous membranes and the surface morphology of the composite samples after DCB and ENF fracture were observed using a scanning electron microscope (LEICA S440). The samples were vacuum coated with gold using a Balzers sputter coater. All images were taken using an accelerating voltage of 5–10 keV with a magnification between 200 times and 2000 times.

## 4. Conclusions

This work demonstrated that interlaminar toughening of G83C, an aerospace grade CFRC, is possible through incorporating electrospun poly(hydroxyether of bisphenol A) (phenoxy) nanofibrous membrane using a simple one step process. The formation of an inverse phase domain morphology characterised by epoxy globules (~2–5 μm) within the phenoxy-rich continuous phase has been evidenced. Although, these domains were homogeneously scattered and confined within the samples’ surface, their concentration was dependent on the phenoxy weight content. The development of these domains during cure is believed to be responsible for the observed improvements in fracture toughness through complex micromechanisms of failure such as cavitation, voiding or localised shear yielding around the epoxy globules. Furthermore, trans-reactions have been shown to occur in interfacial regions of the phase domain boundaries. It is also proposed that our interlayer method using electrospun nanofibres achieved a synergistic toughening effect through both phase separation and inter-domain etherification at the boundaries.
